# Effectiveness of Cognitive Orientation to daily Occupational Performance over and above functional hand splints for children with cerebral palsy or brain injury: a randomized controlled trial

**DOI:** 10.1186/s12887-018-1213-9

**Published:** 2018-07-31

**Authors:** Michelle Jackman, Iona Novak, Natasha Lannin, Elspeth Froude, Laura Miller, Claire Galea

**Affiliations:** 1 School of Child and Adolescent Medicine, The University of Sydney, Sydney, Australia; 2 Occupational Therapy Department, John Hunter Children’s Hospital, Newcastle, Australia; 3 Cerebral Palsy Alliance Research Institute, The University of Sydney, Sydney, Australia; 4 Alfred Health, La Trobe University, Melbourne, Australia; 5 School of Health Science, Australian Catholic University, Sydney, Australia; 6 School of Health Science, Australian Catholic University, Brisbane, Australia

**Keywords:** Upper limb, Task-specific training, Motor training, Cognition, Orthoses, Goal-directed, Occupational therapy

## Abstract

**Background:**

Functional hand splinting is a common therapeutic intervention for children with neurological conditions. The aim of this study was to investigate the effectiveness of the Cognitive Orientation to daily Occupational Performance (CO-OP) approach over and above conventional functional hand splinting, and in combination with splinting, for children with cerebral palsy or brain injury.

**Methods:**

A multisite, assessor-blinded, parallel, randomized controlled trial was conducted in Australia. Participants (*n* = 45) were randomly allocated to one of three groups; (1) splint only (*n* = 15); (2) CO-OP only (*n* = 15); (3) CO-OP + splint (*n* = 15). Inclusion: age 4–15 years; diagnosis of cerebral palsy or brain injury; Manual Ability Classification System I–IV; hand function goals; sufficient language, cognitive and behavioral ability. Primary outcome measures were the Canadian Occupational Performance Measure (COPM) and Goal Attainment Scale (GAS). Treatment duration for all groups was 2 weeks. CO-OP was provided in a group format, 1 h per day for 10 consecutive weekdays, with parents actively involved in the group. Hand splints were wrist cock-up splints that were worn during task practice. Three individual goals were set and all participants were encouraged to complete a daily home program of practicing goals for 1 h. Analyses were conducted on an intention to treat basis.

**Results:**

The COPM showed that all three groups improved from baseline to immediately post-treatment. GAS showed a statistically significant difference immediately post-intervention between the splint only and CO-OP only groups *p* = 0.034), and the splint only and CO-OP + splint group (*p* = 0.047) favoring CO-OP after controlling for baseline.

**Conclusions:**

The CO-OP Approach™ appeared to enhance goal achievement over and above a functional hand splint alone. There was no added benefit of using hand splints in conjunction with CO-OP, compared to CO-OP alone. Hand splints were not well tolerated in this population. Practice of functional goals, through CO-OP or practice at home, leads to goal achievement for children with cerebral palsy or brain injury.

**Trial registration:**

Registered with the Australian New Zealand Clinical Trials Registry (ACTRN12613000690752) on 24/06/2013.

**Electronic supplementary material:**

The online version of this article (10.1186/s12887-018-1213-9) contains supplementary material, which is available to authorized users.

## Background

Cerebral palsy (CP) and brain injury (BI) can significantly impair a child’s ability to use their hands [1]. Therapeutic modalities to improve hand function have progressed significantly over the past 20 years, and there is now a substantial body of evidence to support task-specific upper limb (UL) training interventions in this population [2, 3]. In clinical practice, usual care includes functional hand splinting to promote functional hand use. We wanted to know, whether or not, “Cognitive Orientation to Occupational Performance (CO-OP)”, a new task-specific intervention for the cerebral palsy and brain injury populations, had any clinical benefits over and above functional hand splinting. There also remains limited empirical evidence regarding whether combining UL therapies has any additive effect [2, 3]. We therefore sought to measure the combined effect.

Functional splints are worn when performing an activity, with the aim of supporting one or more joints to maximize the function of the UL during a task. Within the International Classification of Functioning, Disability and Health (ICF) [4], functional splints are a ‘body function and structure’ and ‘environmental’ intervention which aims to support changes in activities by changing the position of the hand. Functional splints are made from various materials, including, but not limited to, neoprene, Lycra™, thermoplastic or tape. Common examples of functional hand splints are a wrist cock-up splint to assist with cutlery use during meal times, a supination splint to assist with catching a ball or a thumb abduction splint to assist with pencil grasp during handwriting [5]. There are a small number of randomized trials investigating functional hand splints [6–10], although there are wide variations in the type of splints investigated, quality of evidence and reliability of outcome measures used in these studies [11]. Functional splints, like many interventions used with children with CP and BI, are often used in combination with other interventions including task-specific training.

Task-specific training is a term used to describe a group of interventions that involve active use of the UL [3]. In the pediatric neurological population, there is high level evidence to support the use of task-specific training, including approaches such as constraint-induced movement therapy (CIMT) and bimanual training [2, 3, 12]. The Cognitive Orientation to daily Occupational Performance (CO-OP) [13] is another task-specific training option. The CO-OP Approach™ combines both motor learning theories with cognitive approaches [14], and shares many of the key ingredients of task-specific training [15] with the important and unique feature of individual child-led problem-solving and strategy choice. In CO-OP, children set their own therapy goals and are guided to discover and develop their own cognitive strategies for successfully carrying out the goal, through the use of the global problem-solving strategy “goal-plan-do-check” [14]. Children are guided to discover their own successful strategies for carrying out a task, instead of the therapist selecting a successful strategy from task analysis and training task performance, which is the convention in other forms of task-specific training. Once a successful strategy has been discovered by the child, children are encouraged to practice the task consistently, as is done in other task-specific UL approaches, in order to bring about the neuroplastic changes in function that underpin motor learning [16]. CO-OP is conventionally carried out over 12 weekly individual therapy sessions, as per the inventor’s recommendations. The CO-OP Approach™ has been piloted in children with CP and BI [13, 17, 18] although there exists limited high level evidence in this population.

The theoretical underpinnings of splinting and CO-OP are very different. When considered in light of the ICF, CO-OP is directly focused on addressing ‘activities’, through cognitive and training strategies, whilst splinting is focused on addressing the ‘impairment’ with the aim of improving function. It is therefore important to explore which of these interventions is most effective, and whether or not there is benefit to combining such interventions. There are currently three different theories that seek to explain the relationship between functional splints and task-specific training. One theory is that a functional splint will allow the user to carry out a task more effectively immediately, with a carry-over effect once the skill is learnt and the splint removed. Another theory is that a functional splint will in fact inadvertently hinder active movement of the limb during task practice, which is vital in the motor learning process. Finally, the “orthotic effect” theory, where the splint is considered to have a neutral effect on motor learning and improved function. The splint improves function when donned, but does not facilitate learning, nor does it inhibit learning. All three theories are currently untested.

The aim of this randomized controlled trial (RCT) was to investigate whether the CO-OP Approach™ led to greater achievement of goals for children with CP or BI over and above conventional splinting alone or when used in combination. The hypotheses for this trial were (1) Children with CP or BI who received CO-OP combined with a splint will achieve comparable improvements in goal achievement and hand function when compared to children who receive CO-OP alone, (2) Children with CP or BI who participate in CO-OP alone will achieve clinically significant changes in goal achievement when compared to children who receive a splint alone.

Our study rationale was that historically therapists sought to induce functional goal achievement using ‘impairment’ interventions (e.g. splinting), whereas newer paradigms preferentially recommend ‘activities’ interventions (e.g. CO-OP or task-specific training). Our hypotheses sought to examine the relative effectiveness of these two different paradigms within the same study.

## Method

### Design and sample size

We conducted a single-blinded RCT that was registered with the Australian New Zealand Clinical Trials Register (ACTRN12613000690752). Detailed study procedures have been previously published [19]. Sample size power calculations were estimated from a previously published 3-group RCT using the same population and outcome measures [20]. We sought an effect size of 0.9, which required 15 participants per each of the three groups, to produce an 80% probability of detecting a 2-point clinically significant change on the 10 point Canadian Occupational Performance Measure [21] (COPM) scale. Statistical significance was set at *p* < 0.05.

### Participants

Children were eligible to participate if they met the following inclusion criteria: (a) Age 4 to 15 years, (b) Diagnosis of CP or BI (minimum 12 months post-injury), (c) Manual Ability Classification System (MACS) I–IV, (d) Impaired hand function as a result of the neurological condition, (e) Child-set goals focused on improving hand function, (f) Sufficient language, cognitive and behavioral skills to set goal topics using the COPM, interact with therapist and participate within a group context (according to CO-OP guidelines), (g) Parents able to commit to a 2 week block of therapy. Exclusion criteria: (a) Impaired hand function resulting from secondary condition (e.g. fracture), (b) Significant intellectual or language impairment (CO-OP guidelines), or (c) Known allergy to splinting materials.

### Procedures

Ethical approval was granted from participating organizations and the University of Notre Dame, Australia. Participants were recruited to this multicenter study through tertiary children’s hospitals and community agencies across three states of Australia from 2013 to 2016. Potential participants were initially screened via email and phone contact. Those deemed likely to be eligible were invited to attend an eligibility assessment. Prior to full baseline assessment, study procedures were fully explained and written consent obtained from the carers of all participants. Participants were randomized immediately following baseline assessment. The randomization sequence was generated using a computer random number generator, with concealment of group undertaken using sequentially numbered and sealed opaque envelopes, stored and opened by an independent offsite officer. To assign group allocation, the principal researcher telephoned the independent officer, who opened the envelope and advised on the assigned group. Blinding of subjects and therapists was not possible due to the nature of the treatment. Masked assessment was carried out at baseline, immediately following the 2 weeks of treatment (primary endpoint) and 8 weeks following completion of treatment by a qualified occupational therapist masked to group allocation. Participants were not provided with previous COPM scores at re-assessment. Data entry was conducted by an independent person masked to group allocation.

### Intervention

Participants were randomly allocated to one of three treatment groups: (1) functional hand splint only, (2) CO-OP only, or (3) CO-OP + a functional hand splint. The total duration of the treatment for all groups was 2 weeks. Each participant’s individual goals, identified on the COPM, were the focus of therapy. All participants were encouraged to complete 1 h of daily home practice of goals, recorded in a logbook. Detailed information regarding the interventions are available in the study protocol [19].

#### Functional hand splinting

All the functional hand splints were a wrist cock-up splint fabricated in either thermoplastic or neoprene with a static insert on the volar surface to support the wrist and block wrist flexion. The prescriber aimed to position the wrist in approximately 20–30° of wrist extension as per splinting conventions, however if this negatively impacted on the individual’s ability to actively extend their fingers and/or functionally grasp, the splint was fabricated in their maximum possible wrist extension with full finger extension. An additional support at the thumb or for supination was included, depending on the child’s hand function and individual goals. To improve wearing tolerance, child and family preference of material were considered. Participants allocated to splint groups were instructed to wear the splint during goal practice (1 h each day), although practice with and without the splint was recorded. Participants in the splint only group were instructed to practice goals at home and did not undertake any face-to-face intervention with a therapist.

#### Cognitive Orientation to daily Occupational Performance (CO-OP)

A total of 10 sessions were carried out over 10 consecutive weekdays, for approximately 1 h per session, within the clinic environment. This study aimed to adhere to the critical components of CO-OP, and CO-OP fidelity checklists [22] were utilized to ensure that CO-OP was being provided and not some other task training. The study aimed to provide CO-OP training to participants within a small group (3–4 children). Due to recruitment numbers and randomization sequence factors, the groups varied in the number of participants (range 2–5) depending on recruitment rates at that site. This meant some participants needed to receive individual CO-OP intervention (*n* = 6) because they were a “group of one”. Parents were active participants within the sessions.

#### Functional hand splinting + CO-OP

Participants randomized to the CO-OP + splint group undertook CO-OP, whilst being prescribed with a hand splint, as described above. Children were expected to wear their splint at all times during practice of goals, both within the CO-OP group and during home practice. Logbooks recorded time spent with the splint on and off during goal practice. In line with ethical considerations, if a child did not assent to wearing the splint their wishes were respected. Researcher and parent notes were taken regarding reasons the child chose to discontinue wearing the splint.

### Outcome measures

All outcome measures collected are reported in this paper. Outcome measures were collected at baseline, immediately following the 2 weeks of intervention, and primary outcome measures only were collected at 8 weeks post intervention (follow up).

#### Primary outcome measures

Primary outcome measures were the COPM [21] and Goal Attainment Scale (GAS) [23], with the study powered to detect a change on the COPM. The COPM is the ICF activities level recommended tool of choice when using CO-OP, according to the developer of CO-OP’s recommendations [14]. COPM is a standardized goal setting and outcome measurement tool commonly used in pediatric rehabilitation practice and research [21, 24] and is validated for both child report and parent proxy report. The COPM enables the participant to rate their performance, as well as satisfaction on a scale of 1–10 for each individual goal. As per the COPM administration manual, children who were able to understand the concept of rating the COPM scored themselves. Whereas if the child had difficulty understanding the numeracy concepts of scoring, parents completed the COPM proxy-scoring, which is known to be valid, reliable and responsive in young children with cerebral palsy [25]. For example, children with intellectual disability or children younger than 8 years old. Whoever rated the COPM at baseline assessment also rated at follow up assessment. COPM raw scores (range 0–10) were used to determine change.

The GAS is a standardized measure of goal achievement that measures change in an individuals goals [23], according to a five point scale, in which − 2 is the current level of function, 0 is the expected level of function and + 2 far exceeds the expected level of function following the treatment. GAS scores were not weighted. Data analysis utilized GAS T-scores (range 22–78).

#### Secondary outcome measures

Secondary outcome measures included the Box and Block Test [26] (BBT) and wrist range of motion (ROM), which are ICF body structures level measures that reflect the therapeutic intent of splinting. The BBT is an assessment of grasp and release, in which the participant transfers individual blocks from one side of a box, over a partition to the other side, over a 60s period [26]. The score is the total number of blocks moved (range 0–150). A number of studies have utilized the BBT for children with CP [27–29], although reliability and validity in this population is unclear. Strong test-retest and interrater reliability has been shown in typically developing children [26, 30].

Wrist ROM comprised of passive wrist ROM (with fingers flexed), Volkmann’s angle [31] (with fingers extended) and active wrist ROM (with fingers flexed). An external wrist ROM device was utilized to standardize ROM measurements in an effort to improve interrater reliability. Joint angle was measured using a digital inclinometer, with change measured in whole numbers of degrees (range 0–180).

### Statistical analysis

Participant characteristics were analyzed using descriptive statistics. A one-way ANCOVA controlling for COPM performance at baseline was also conducted to ensure no significant baseline differences between all three groups. All data were assessed for normality using Shapiro-Wilks and visual inspection of boxplots. All analyses were conducted on an intention to treat basis, as per the study protocol. Statistical significance was set at *p* < 0.05 (two-tailed). Two-way mixed ANOVA with repeated measures were undertaken to account for expected correlation within participant scores over the three time points. ANCOVA controlling for baseline score were conducted when only two time points were used. Where there was contamination between the treatment groups, i.e. participants deviated from the treatment protocol, post-hoc secondary analyses on primary outcomes were run using the same analysis methodology as intention to treat. All data were analyzed using SPSS (V.24) and STATA (STATA, Version 14, StataCOrp, College Station, TX, USA). Findings are reported according to the CONSORT statement [32].

## Results

**Fig. 1:**
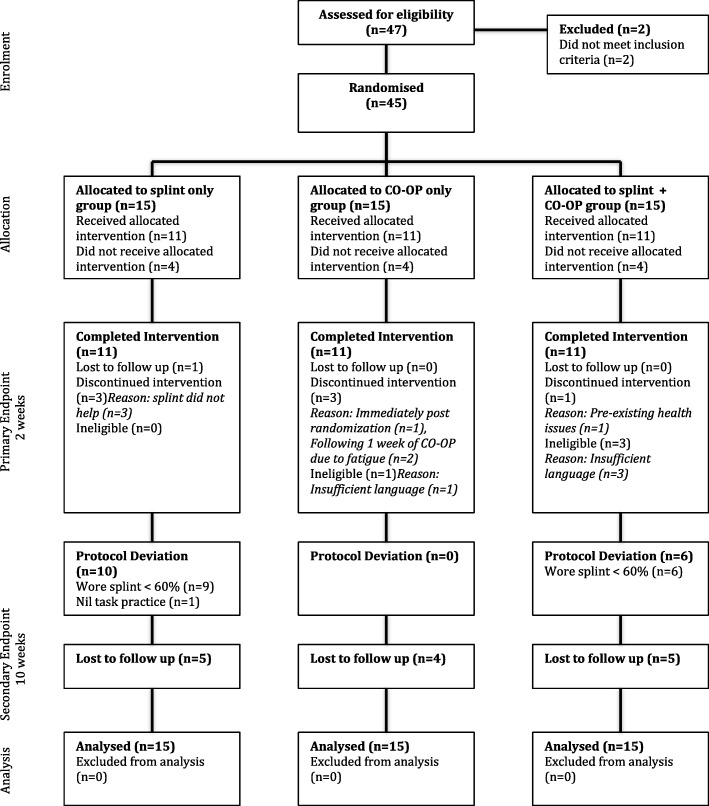
CONSORT diagram of flow of participants through trial. Legend: Deviation based on 60% adherence to protocol

A total of 45 children (22 females and 23 males) were randomized to the three intervention groups. Participant flow is shown in Fig. 1.

Participants ranged from 4.1 to 15.2 years, MACS I–IV and GMFCS I–V. Participant baseline characteristics are shown in Table 1. Overall, 33 of the 45 participants completed the intervention (Splint only group *n* = 11[73%], CO-OP only group *n* = 11[73%], CO-OP + splint group *n* = 11[73%]). The only variable different between the groups at baseline was unilateral impairment topography. Analyses were conducted using the MACS classification, which is known to be more objective and stable.

**Table 1:** Baseline characteristics of participants

Participant Information	Whole Sample *n* = 45	Group 1 (Splint only) *n* = 15	Group 2 (CO-OP only) *n* = 15	Group 3 (CO-OP + Splint) *n* = 15
Age (mean (SD))	8.4 (2.7)	8.3 (2.8)	8.1 (2.3)	8.8 (3.1)
Gender				
Male (*n*, %)	23 (51)	8 (53)	8 (53)	7 (47)
Female (*n*, %)	22 (49)	7 (47)	7 (47)	8 (53)
Diagnosis				
Cerebral Palsy (*n*, %)	40 (89)	13 (87)	15 (100)	12 (80)
Brain injury (*N*, %)	5 (11)	2(13)	0	3 (20)
Limbs affected				
Unilateral (*n*, %)	32 (71)	14 (93)	11 (73)	7 (47)^a^
Bilateral (*n*, %)	13 (29)	1 (7)	4 (27)	8 (53)
Motor Type				
Spastic (*n*, %)	28 (62)	9 (60)	11 (73)	8 (53)
Dystonic (*n*, %)	5 (11)	2 (13)	0	3 (20)
Mixed (*n*, %)	11 (24)	4 (27)	3 (20)	4 (27)
Ataxic (*n*, %)	1 (2)	0	1 (7)	0
MACS				
I (*n*, %)	5 (11)	3 (20)	1 (7)	1 (7)
II (*n*, %)	28 (62)	10 (67)	10 (67)	8 (53)
III (*n*, %)	9 (20)	2 (13)	3 (20)	4 (27)
IV (*n*, %)	3 (7)	0	1 (7)	2 (13)
GMFCS				
I (*n*, %)	24 (53)	9 (60)	8 (53)	7 (47)
II (*n*, %)	10 (22)	4 (27)	4 (27)	2 (13)
III (*n*, %)	4 (9)	1 (7)	0	3 (20)
IV (*n*, %)	6 (13)	1 (7)	3 (20)	2 (13)
V (*n*, %)	1 (2)	0	0	1 (7)
House				
1 (*n*, %)	20 (44)	6 (40)	7 (47)	7 (47)
2 (*n*, %)	4 (9)	2 (13)	0	2 (13)
3 (*n*, %)	14 (31)	4 (27)	7 (47)	3 (20)
4 (*n*, %)	3 (7)	1 (7)	0	2 (13)
No contracture	4 (9)	2 (13)	1 (7)	1 (7)
COPM score, mean (SD)				
COPM-P	2.75 (1.34)	2.78 (1.32)	2.16 (0.98)	3.32 (1.48)
COPM-S	3.38 (1.47)	3.37 (1.43)	3.07 (1.38)	3.69 (1.62)

Legend: *MACS* Manual Ability Classification System, *GMFCS* Gross Motor Function Classification System, *House* House thumb classification, *COPM-P* Canadian Occupational Performance Measure Performance Score, *COPM-S* Canadian Occupational Performance Measure Satisfaction Score; ^a^ Statistically different at baseline

### Primary outcomes

#### COPM

All groups improved on both COPM performance and COPM satisfaction scores from baseline to immediately post-intervention (Table 2). There were no statistically significant differences between the three groups immediately following the intervention (after controlling for baseline) or 8 weeks post-intervention (repeated measures) on COPM Performance or COPM Satisfaction, as shown in Table 3. Between-group intervention contamination occurred, as children abandoned their splints, preferring to carry out goal practice splint free (refer to dose of practice section).

**Table 2:** Results at baseline, immediately following treatment (2 weeks), and at follow up (10 weeks)

Outcome measure	Outcome Score		
	Splint only	CO-OP only	CO-OP + Splint
COPM-Per Mean(SD)			
Baseline	2.78 (1.32)	2.16 (0.98)	3.32 (1.48)
Immediate	5.43 (2.12)	5.89 (2.37)	6.11 (2.43)
Follow up	5.41 (2.00)	5.36 (2.21)	6.33 (2.05)
COPM–Sat Mean(SD)			
Baseline	3.37 (1.43)	3.06 (1.38)	3.69 (1.62)
Immediate	5.78 (2.20)	6.47 (2.26)	6.34 (2.32)
Follow up	5.88 (2.19)	6.51 (2.21)	6.33 (2.19)
GAS, Mean (SD)			
Baseline	22.79 (0.67)	22.61 (0.0)	22.79 (0.67)
Immediate	39.24 (9.95)	50.91 (14.14)	50.41 (18.89)
Follow up	39.24 (15.26)	49.02 (14.53)	49.92 (17.18)
BBT, Mean (SD)			
Baseline	12.1 (10.2)	12.4 (10.6)	11.5 (11.0)
Immediate	12.1 (10.7)	14.5 (11.9)	12.6 (11.8)
Wrist Extension PROM, degrees (Mean, SD)			
Baseline	53.4 (25.2)	60.8 (21.1)	60 (29.5)
Immediate	59.6 (21.4)	64 (21)	63.5 (24.5)
Wrist Extension AROM, degrees (Mean, SD)			
Baseline	19.9 (42.4)	23.4 (35.1)	37.1 (37.4)
Immediate	30.1 (35.1)	28.6 (32.2)	40.5 (31)
Volkmann’s angle, degrees (Mean, SD)			
Baseline	36.9 (38.1)	43.2 (34.8)	37.7 (47.3)
Immediate	40.7 (38.2)	37.9 (50.1)	32.7 (54.3)

Legend: *COPM-Per* Canadian Occupational Performance Measure – Performance, *COPM-Sat* Canadian Occupational Performance Measure – Satisfaction, *GAS* Goal Attainment Scale, *BBT* Box and blocks test, *PROM* Passive range of motion, *AROM* Active range of motion. *P* value significance set at *p* < 0.05

**Table 3:** Intention to treat (ITT) Results and between group ANCOVA analyses immediately following treatment (2 weeks), and repeated measures at follow up (10 weeks). Analyses used are specified within table

Outcome Measure	Group	Estimated Mean	Estimated 95% CI	*P* value
Repeated measures analysis (3 Time points) *n* = 45				
COPM PER	Splint	4.54	3.72–5.36	*p* = 0.052
	COOP	4.47	3.65–5.29	
	CO-OP + Splint	5.25	4.43–6.07	
COPM SAT	Splint	5.02	4.15–5.88	*p* = 0.756
	COOP	5.35	4.48–6.21	
	CO-OP + Splint	5.45	4.59–6.32	
GAS	Splint	33.76	28.75–38.76	*p* = 0.072
	COOP	40.85	35.84–45.86	
	CO-OP + Splint	41.04	36.03–46.05	
ANCOVA (Controlling for baseline 2 time points pre and immediately following intervention)				
COPM PER	Splint	5.41	4.24–6.59	Splint – COOP *p* = 0.371
	COOP	6.17	4.95–7.40	Splint – CO-OP + splint *p* = 0.618
	CO-OP + Splint	5.83	4.62–7.05	COOP – CO-OP + Splint *p* = 0.702
COPM SAT	Splint	5.79	4.70–6.89	Splint – COOP *p* = 0.268
	COOP	6.66	5.55–7.76	Splint – CO-OP + Splint *p* = 0.647
	CO-OP + Splint	6.15	5.04–7.25	COOP – CO-OP + Splint *p* = 0.518
GAS	Splint	39.12	32.23–46.91	Splint – COOP *p* = 0.034
	COOP	51.16	43.32–59.00	Splint – CO-OP + Splint *p* = 0.047
	CO-OP + Splint	50.29	42.50–58.08	COOP – CO-OP + Splint *p* = 0.875
BBT	Splint	11.97	10.55–13.40	Splint – COOP *p* = 0.047
	COOP	14.02	12.60–15.45	Splint – CO-OP + Splint *p* = 0.250
	CO-OP + Splint	13.14	11.71–14.57	COOP – CO-OP + Splint *p* = 0.382
PROM^a^	Splint	62.00	52.21–71.59	Splint – COOP *p* = 0.893
	COOP	60.94	50.57–71.32	Splint – CO-OP + Splint *p* = 0.932
	CO-OP + Splint	62.48	52.84–72.13	COOP – CO-OP + Splint *p* = 0.827
WROM^a^	Splint	34.35	22.93–45.76	Splint – COOP *p* = 0.482
	COOP	28.50	16.29–40.70	Splint – CO-OP + Splint *p* = 0.661
	CO-OP + Splint	30.64	18.29–42.99	COOP – CO-OP + Splint *p* = 0.804
Volkmann’s angle^a^	Splint	42.25	21.90–62.60	Splint – COOP *p* = 0.461
	COOP	31.25	9.36–53.14	Splint – CO-OP + Splint *p* = 0.550
	CO-OP + Splint	33.69	13.32–54.01	COOP – CO-OP + Splint *p* = 0.871

Legend: *COPM-Per* Canadian Occupational Performance Measure – Performance, *COPM-Sat* Canadian Occupational Performance Measure – Satisfaction, *GAS* Goal Attainment Scale, *BBT* Box and blocks test, *PROM* Passive range of motion, *AROM* Active range of motion. *P* value significance set at *p* < 0.05

^a^PROM, WROM and Volkmann’s angle –ANCOVA results should be interpreted with caution model fit poor

Of the 45 participants who were enrolled into the study, 26 participants were able to score the COPM independently. Nineteen participants were unable to independently score the COPM. Of these 19, three children scored the COPM with assistance from a parent and in the other 16 cases the parents scored the COPM for the child. The reason for a parent needing to score the COPM was primarily age (*n* = 10 participants were under 6 years of age). Children over 6 years of age were given the opportunity to determine their own scores on the COPM, however the assessing therapist, in conjunction with the parent, made a decision regarding the participant’s ability to rate the COPM independently. Reasons for children over the age of six requiring parental assistance included cognitive delay (*n* = 5), attention deficit (*n* = 2) and language delay (*n* = 2) (Additional file 1: Table S1).

#### GAS

For GAS scores, there was a statistically significant difference between the splint only and the CO-OP only groups (*p* = 0.034) as well as the splint only and CO-OP + splint (*p* = 0.047) immediately post-treatment, in favor of the CO-OP group. Analyses indicated a type II error for GAS data, suggesting that the study was underpowered. GAS data, as shown in Table 2 suggested a trend towards greater improvements in the CO-OP only and CO-OP + splint groups compared to the splint only group.

### Secondary outcomes – BBT and ROM

There was a statistically significant within group difference between splint only and CO-OP only (*p* = 0.047) immediately post-treatment in favor of CO-OP only. There were no other statistically significant between group differences after controlling for baseline (Table 3).

### Dose of practice

Information regarding dosage of task practice and splint wear is detailed in Table 4. At study commencement, all three groups were instructed to practice tasks at home at the exact same dosage, and the two splint groups (Groups 1 & 3) were instructed to wear the splints during the home practice for the same dosage. However, not all participants adhered to the prescribed dosage for splint-wearing or task practice at home. For both the CO-OP groups (Groups 2 & 3), CO-OP was provided face-to-face, in addition to the home practice with or without splint wearing depending on group allocation. The mean (SD) dose of the home-based task practice for each group in minutes, self-selected by participants, was: Splint only = 353 (186); CO-OP only = 856 (438); CO-OP + Splint = 893 (450). In the splint only group, participants adhered to the prescribed splint wearing on average 47.1% of the expected prescribed minutes, and in the CO-OP + splint group, participants adhered to the prescribed splint wearing on average 47.3% of the expected prescribed minutes. One participant in the CO-OP + splint group withdrew due to ill health arising from a pre-existing medical condition, which is a known confounder in childhood disability trials.

**Table 4:** Dosage of intervention (time in minutes)

DOSE	Splint only (*n* = 11)	CO-OP only (*n* = 13)	CO-OP + Splint (*n* = 13)
Total dosage of task practice, minutes			
Mean (SD)	353(186)	856 (438)	893 (450)
Range	40–600	300–1680	240–1860
Dosage of CO-OP, minutes			
Mean (SD)	N/A	485 (111)	466 (139)
Range		300–600	180–600
Dosage of task practice at home, minutes			
Mean (SD)	330 (188)	372 (382)	427 (398)
Range	40–600	0–1080	60–1320
Time splint worn, minutes			
Mean (SD)	174 (157)	N/A	459 (421)
Range	0–450		30–1440
% time splint worn during task practice			
Mean %	47.1%	N/A	47.3%
Range %	0–100%		3.6–100%

*N/A* Not applicable

At completion of the study, children and parents were asked “If given the choice would you have worn the splint during practice of goals?”, to which 64% (16/25) responded no. Reasons given by children and parents included: the splint restricted movement, making it difficult to grasp and release; the splint made practice of goals more difficult; and the splint was poorly tolerated by the child.

Per protocol post hoc secondary analyses were run and no additional statistically significant between group differences were identified with dropouts removed (Additional file 1: Table S2).

## Discussion

In this three group, pragmatic RCT, all groups showed statistically significant within-group improvements following 2 weeks of treatment. Between-groups, goal attainment was greater for those who received CO-OP, compared to a functional hand splint and practicing goals at home. Combined use of CO-OP and splinting had no additive effect over CO-OP alone. Splints were not well tolerated by our participants, and participants deviated from the protocol by practicing goals without the splint on. The dose of task practice required to achieve significant improvements in this study was much lower than suggested minimum UL task-specific training dosage [3]. CO-OP, as well as task-specific practice of goals at home, may be effective interventions that lead to goal achievement when collaborative client-centered goals are set, a short, intense block of therapy is prescribed and the treatment is focused on active practice of the child’s chosen goals.

We investigated whether CO-OP added any benefits over and above a functional hand splint alone and when used in combination. Children provided with CO-OP in addition to splint demonstrated no greater improvement in goal achievement than children who completed CO-OP alone. In contrast to our findings, Elliott and colleagues [7] found that children who received a splint plus goal-directed training improved more on GAS scores than children who completed goal-directed training alone. Further research may be needed, however given the poor tolerance of splints in our study it may be ethically challenging to justify a larger trial of this nature.

CO-OP was shown to lead to goal achievement, therefore may be another beneficial task-specific training option for children with CP or BI. Task-specific UL training approaches, that involve active practice of a task, rather than addressing underlying impairments, are now widely recognized as best practice in this population [33]. CO-OP may be utilized with children who are able to set their own goals, have the communication and cognitive skills to problem-solve and are motivated to persist with practice of goals. CO-OP can be used with children with unilateral or bilateral impairment, with a range of functional abilities. In other populations, CO-OP has been shown to have the additional benefit of transfer of problem-solving skills to future goals and functional skills [34]. We did not investigate transfer of skills, although a study of CO-OP for children with BI suggested transfer may not be achieved [18], warranting further investigation. As CO-OP is a promising intervention in this population, there is a need to provide CO-OP training to therapists in an effort to translate this new evidence into clinical practice.

Splints were not well tolerated by children in our study and this in itself is an important finding. Dislike of splint wearing and self-selected abandonment has been observed in other clinical populations [35–37]. Participants in the splint group, who were expected to practice their goals daily with their hand splint on, generally chose not to wear their splint, but instead to practice their goals without the splint on. The majority of children who were provided with a splint chose to wear it less than 50% of the time during goal practice, despite instructions to wear the splint 100% of the time. It appears that if participants did not find the splint useful, it was discarded and they continued to practice goals without the splint. Intervention contamination between-groups therefore occurred, and the splint only group were completing goal-directed, task-specific training at home. In doing so, these participants were able to achieve their goals, suggesting children may have discerned what was working, and thus were motivated to practice using the effective goal-direct task-specific training strategy. Daily, targeted practice of goals within the home may be another effective task-specific training option, consistent with previously reported benefits of home programs [20]. The lack of difference between the groups was therefore not surprising given the number of children in the splint only group that did not adhere to the protocol, and instead carried out task-specific training in a home program format, which has similarities with the CO-OP approach. Previous head-to-head trials of different types of task specific training for children with cerebral palsy (e.g. Constraint Induced Movement Therapy versus Bimanual Training), have showed no differences in outcomes between types of task-specific training interventions [2, 3]. It is interesting that the children performing home programs achieved similar outcomes on the COPM, because home programs provide a low-cost alternative with no travel requirements for parents. Previous splinting studies have reported poor tolerance of external garments by children [38] and static splints by adults [35–37], however there have also been studies that have reported no issues with splint tolerance [7, 8, 38].

Dose, or total amount of practice has been identified as an essential consideration in task-specific training [32]. Previous studies have suggested a minimum of 40 h of practice may be required to achieve significant improvements in UL function [3]. In our study, the dose of practice was much less than this suggested minimum dosage, consistent with previous CO-OP studies in the developmental coordination disorder population [14, 33]. The splint only group improved with an average of approximately 6 h of self-selected goal practice at home and the CO-OP groups improved with approximately 14 h of practice over a 2 week period (10 h face-to-face plus 4 h at home) (Table 3). Possible explanations for these positive results from lower dose intervention include: (a) the interventions in this study focused solely on practice of the three goals as chosen by each individual child, whereas in other cerebral palsy and brain injury studies [3, 12], participants may practice many tasks that target improved upper limb function. It is possible that a smaller dose of practice, such as the 6–14 h achieved in our study, is enough to successfully achieve three individual goals, whereas a larger dose, for example 40 h, is required in order to not only achieve goals, but also to improve hand function as measured on the Assisting Hand Assessment (AHA) and Quality of Upper Extremity Skills Test (QUEST) [3]; (b) CO-OP is more effective than other task specific approaches at low doses in the cerebral palsy population, because CO-OP teaches a global problem solving strategy that the child can use to solve problems at home when the therapist is not present [13]. The only previous study of CO-OP in the cerebral palsy population found that CO-OP led to greater generalization, supporting this proposition [13]; (c) In regard to outcome measures, the COPM and GAS, which measure changes in ‘activities and participation’ were of interest in this study, in keeping with the ICF focus in pediatric rehabilitation and newer theories of motor learning. In our study the primary outcome measures were the COPM and GAS, whereas previous task-specific training studies have utilized assessments such as the AHA or QUEST in combination with goal achievement outcomes. The COPM and GAS are known to be highly responsive to detecting small individualized gains. The differences between the COPM and GAS outcomes are not clear, however one theory is that the COPM is more subjective than the GAS. It has previously been suggested that participants are likely to perceive whichever therapy they receive as effective, and this may be reflected in COPM outcomes. The GAS may be more objective, and therefore may be more likely to reflect actual improvements in goal achievement, rather than perceived improvement. Moreover, although the BBT provided a basic measure of hand function, it is understandable that children did not improve on the BBT as a result of CO-OP as they did not practice grasping and releasing blocks as part of their treatment. Participants practiced their own goals and therefore we wanted to measure if those goals had been achieved; and (d) Undertaking CO-OP with a therapist face-to-face where motivation and the “just right challenge” for learning is implemented, as opposed to prescribing a splint with self-directed practice at home, perhaps is more likely to lead to a greater dose of training and therefore a better outcome.

### Future directions

The results of our study further support the benefits of task-specific training approaches in various forms for children with CP or BI. Further research comparing CO-OP or task-specific home programs to proven task-specific training approaches, such as CIMT or bimanual training is warranted, particularly as dose requirements appear lower enabling cost effective services. Further research is needed regarding the types of children with CP or BI who may respond best to the CO-OP Approach™. A larger sample would enable sub-group analyses by etiology and type of cerebral palsy and brain injury, which would provide valuable information to clinicians about responders. Education regarding CO-OP is needed for therapist working with children with cerebral palsy and brain injury in order to translate this new evidence into practice in this population. Future studies should plan to recruit a much larger sample size, based on a power calculation using this new pilot data.

### Limitations

This was a pragmatic trial that had small numbers and included a very broad population in regard to age, diagnosis and motor abilities, this is a study limitation. There are several other limitations to this pilot study, and the results must be interpreted cautiously. First, there were a large number of withdrawals in each group, and a number of participants who deviated from the study protocol (Fig. 1). It is possible that children who may benefit from CO-OP differ from those who may benefit from functional hand splints. Pre-trial participant treatment preferences may have biased recruitment and adherence. Poor splint wearing adherence, affected the statistical power for both the between-group analysis and dose response analysis. Second, it is difficult to prescribe one splint that is suitable for three goals, each of which may require a different hand position. It is possible that poor design of the splint led to poorer hand function, although measures were in place to limit this possibility. Block randomization would have been beneficial in order to facilitate homogeneous CO-OP groups. Third, the comparison of CO-OP in center-based group format, to individualized splint-wearing at home, introduces another confounder that may explain the study results. Fourth, the use of a self-reported goal-based measure as a primary end-point rather than an objective hard end-point measure, may have influenced the results. Fifth, the combined use of child self-reporting and parent proxy-reporting of the primary end-point measure (COPM) may have influenced the results. Sixth, contamination of trial groups led to small sample sizes for regression analysis. Cautious interpretation of the results is therefore recommended.

## Conclusion

Task-specific training continues to be best practice in supporting goal achievement for children with CP or BI, with CO-OP being a new form of task-specific training useful in these populations. CO-OP or task-specific training at home may be intervention options that require a lower dose to achieve individual goals, although CO-OP because of its structured problem-solving approach is likely to be more effective than child-led home practice alone. Combined use of CO-OP and functional hand splints did not lead to any additional benefits over CO-OP alone, and splints were not well tolerated by our participants. Therapy can be maximized through child-chosen goals, setting short, intensive timeframes and treatment including active practice of goals.

## Additional file

Additional file 1: **Table S1.** COPM Ratings. **Table S2.** Excluding dropouts results and between group ANCOVA analyses immediately following treatment (2 weeks), and repeated measures at follow up (10 weeks). Analyses used are specified within table. (DOCX 31 kb)

## Abbreviations

AHA: Assisting hand assessment
BBT: Box and Blocks Test
BI: Brain injury
CIMT: Constraint-induced movement therapy
CO-OP: Cognitive Orientation to daily Occupational Performance
COPM: Canadian Occupational Performance Measure
CP: Cerebral palsy
GAS: Goal attainment scale
GMFCS: Gross motor function classification system
ICF: International Classification of Functional, Disability and Health
MACS: Manual abilities classification system
QUEST: Quality of Upper Extremity Skills Test
RCT: Randomised controlled trial
ROM: Range of motion
UL: Upper limb
