# Supplementary material for: Effectiveness of Cognitive Orientation to daily Occupational Performance over and above functional hand splints for children with cerebral palsy or brain injury: a randomized controlled trial
Source: BMC Pediatr. 2018 Jul 31;18:248. doi: 10.1186/s12887-018-1213-9 (PMC6069709; doi:10.1186/s12887-018-1213-9)
Supplement: Supplementary file 1 — Table S1. COPM Ratings. Table S2. Excluding dropouts results and between group ANCOVA analyses immediately following treatment (2 weeks), and repeated measures at follow up (10 weeks). Analyses used are specified within table. (DOCX 31 kb) [file 12887_2018_1213_MOESM1_ESM.docx]

Additional file 1

| **Participant number** | **Who rated COPM** | **Reason** |
| --- | --- | --- |
| 001 | Parent | Age (4 years) |
| 002 | Parent | Age (4 years) |
| 003 | Parent | Age (5 years) |
| 005 | Parent | Age (6) + Autism Spectrum Disorder (Language and behavioral difficulties) |
| 007 | Parent | Age (6) + cognitive impairment |
| 012 | Child with assistance from parent | Cognitive impairment (11 years of age) |
| 017 | Parent | Age (5) |
| 019 | Parent | Age (5) + Autism Spectrum Disorder |
| 021 | Parent | Age (6) + attention issues |
| 022 | Parent | Age (4) |
| 024 | Parent | Age (4) |
| 026 | Parent | Cognitive delay (age 7 years) |
| 027 | Child with assistance from parent | Attention deficit (un-medicated at time of intervention as school holidays) (age 8) |
| 028 | Parent | Age (4) |
| 031 | Parent | Age (4) |
| 033 | Child with assistance from parents | Suspected cognitive delay (age 11) |
| 039 | Parent | Age (5) |
| 040 | Parent | Cognition and language deficit |
| 042 | Parent | Age (5) |

Table S1: COPM Ratings

| Repeated measures analysis (3 Time points) n = 33 | | | | |
| --- | --- | --- | --- | --- |
| Outcome Measure | Group | Estimated Mean | Estimated 95% CI | P value |
| COPM PER | Splint  COOP  CO-OP + Splint | 5.07  4.84  6.07 | 4.46 – 5.67  4.24 – 5.45  5.46 – 6.67 | *p*=0.242 |
| COPM SAT | Splint  COOP  CO-OP + Splint | 5.37  5.64  6.15 | 4.73 – 6.01  5.00 – 6.28  5.51 – 6.79 | *p*=0.097 |
| GAS | Splint  COOP  CO-OP + Splint | 35.89  43.91  46.22 | 31.45 – 40.34  39.47 – 48.36  41.77 – 50.66 | *p*=0.292 |
| ANCOVA (Controlling for baseline 2 time points pre and immediately following intervention) | | | | |
| Outcome Measure | Group | Estimated Mean | Estimated 95% CI | P value |
| COPM PER | Splint  COOP  CO-OP + Splint | 6.37  6.48  7.04 | 5.40 – 7.35  5.48 – 7.49  6.01 – 8.07 | Splint – COOP *p*=0.876  Splint – CO-OP + Splint *p*=0.350  COOP – CO-OP + Splint *p*=0.453 |
| COPM SAT | Splint  COOP  CO-OP + Splint | 6.64  7.09  6.92 | 5.63 – 7.64  6.08 – 8.11  5.87 – 7.96 | Splint – COOP *p*=0.514  Splint – CO-OP + Splint *p*=0.703  COOP – CO-OP + Splint *p*=0.811 |
| GAS | Splint  COOP  CO-OP + Splint | 42.03  55.31  59.04 | 35.06 – 49.00  48.34 – 62.28  51.96 – 66.12 | Splint – COOP *p*=0.010  Splint – CO-OP + Splint *p*=0.002  COOP – CO-OP + Splint *p*=0.453 |
| BBT | Splint  COOP  CO-OP + Splint | 13.92  16.35  14.46 | 12.12 – 15.72  14.56 – 18.14  12.67 – 16.25 | Splint – COOP *p*=0.062  Splint – CO-OP + Splint *p*=0.669  COOP – CO-OP + Splint *p*=0.137 |
| PROM** | Splint  COOP  CO-OP + Splint | 64.21  59.75  61.56 | 52.24 – 76.19  47.19 – 72.30  49.59 – 73.53 | Splint – COOP *p*=0.602  Splint – CO-OP + Splint *p*=0.751  COOP – CO-OP + Splint *p*=0.832 |
| WROM** | Splint  COOP  CO-OP + Splint | 37.51  25.33  31.90 | 24.28 – 50.73  11.48 – 39.18  17.23 – 46.58 | Splint – COOP *p*=0.202  Splint – CO-OP + Splint *p*=0.566  COOP – CO-OP + Splint *p*=0.510 |
| Volkmann’s angle** | Splint  COOP  CO-OP + Splint | 41.47  23.37  21.47 | 15.73 – 67.22  -3.61 – 50.34  -4.26 – 47.20 | Splint – COOP *p*=0.329  Splint – CO-OP + Splint *p*=0.270  COOP – CO-OP + Splint *p*=0.918 |

Table S2: Excluding dropouts results and between group ANCOVA analyses immediately following treatment (2 weeks), and repeated measures at follow up (10 weeks). Analyses used are specified within table.

Legend: COPM-Per = Canadian Occupational Performance Measure – Performance; COPM-Sat = Canadian Occupational Performance Measure – Satisfaction; GAS = Goal Attainment Scale; BBT = Box and blocks test; PROM = Passive range of motion; AROM = Active range of motion. P value significance set at p < 0.05.

**PROM, WROM and Volkmann’s angle –ANCOVA results should be interpreted with caution model fit poor
